# Yellow Fever, a More Indigenous Than Exotic Disease and Not Belonging to the Zymotic in the Shape of Contagion

**Published:** 1880-06-20

**Authors:** L. V. Weathers

**Affiliations:** Ark.


### YELLOW FEVER, A MORE INDIGENOUS THAN EXOTIC DISEASE AND NOT BELONGING TO THE ZYMOTIC IN THE SHAPE OF CONTAGION.

BY L. V. WEATHERS, M.D., OF ARK.

You will please allow me to notice through your columns the article of Dr. Greenley, of Kentucky, upon the nature of yellow fever and its fearful ravages. The Doctor takes the position that it is indigenous, the only man whom I have read after through any of our journals who really advocated what I believe to be the true and chief cause of this malady.

I believe yellow fever to be based upon malarial influences alone, the same causes giving rise to some of our most malignant and pernicious types of fever, being in a more poisonous form still, when confined to crowded towns and cities, being mixed with the animal as well as decayed vegetable matter, which being pent up in the walls of large cities and towns becomes more poisonous in its nature. The inhabitants all breathe in the same atmospheric poison; all become infected from the same cause, and consequently greater is its spread, and hence, on this account alone, it is now classed as zymotic, propagated by contagion or contact. I believe the germ of yellow fever lives in cities built upon low flat ground, for ages, and even while building cities the uneven ground is often filled by rubbish, etc., which may form by decay new earth, and the dampness, which may originate in cellars or around cities, arises oftentimes from new made earth of vegetable and animal matter.

I read a report from one of the Sanitary Committees of New Orleans some two years ago, and it was discovered that the dump carts had emptied loads of refuse matter, animal and vegetable, in portions of the city until they had filled some ravines or uneven spots of ground, and upon a careful investigation it was found to be nothing more than a soft pulpy mass from the animal and vegetable decay going on beneath, a poison emanating from rotten cabbage, etc. Is not this malarial and animal poison combined? And is not this the case in every city without the aid of vigilant sanitary committees?

Then I concur with Dr. Greenley that it is not an exotic disease but has its nidus among us. The Doctor says where there are so many different views and opinions regarding any one thing, it is pretty strong proof that it is either poorly understood, or that the subject is obscure and exceedingly difficult to investigate. I heartily endorse him in this position, and at the same time maintain my position that it is a malarial disease and generated by malarial influences, and like our pernicious fevers, frost puts an end to it. I cannot but think that our so-called swamp fever here, or in other words yellow fever, for it is nothing else in its true sense in a modified form, all of which is based upon malarial influences, confined, as its name designates, to low, flat swamps, the nests of malaria, and if this same cause mixed with animal matter such as they have in cities, and that atmosphere pent up, it would have all the malignancy of yellow fevers that occur in cities. I say so without fear of successful contradiction. I meet with yellow fever here without all these contaminating surroundings, and what do we see: the patients, inhabitants of malarial districts, having chills for awhile previous; finally, a distinct rigor seizes the patient; severe and constant vomiting, with discharge of ropy mucus, ensues; now and then a little green bile, but seldom; the straining continues, and retching to a distressing degree; the urine now is nothing but pure blood, the bowels closely confined, and the patient as yellow as a pumpkin—even the eyes perfectly orange.

What is this but yellow fever based upon malaria?—and the odor which emanates from the body is really sickening.

I have seen these cases, and treated them successfully in the following way, and had I a regular case of yellow fever in Memphis or New Orleans, I would treat it upon the same principle and would risk my life on it if I was its victim.

*Treatment.*—If you see the victim early, give four or five grains of calomel with opium if you can, but I had rather rely upon the calomel alone. You cannot give anything by the stomach after twenty-four hours. Use boiled corn around the patient; keep twenty-four ears in a large pot, place twelve ears around the patient and continue the heat until he sweats freely; then rub the inside of the thighs with mercurial ointment, and bathe the body every two hours, and rub it in with quinine, strong tincture of capsicum, and good apple vinegar.

This is the only treatment, and when you succeed in salivation you then can claim your patient safe, and not 'till then; and not a severe salivation are you going to set up after all. Does not this show the re-establishment of the secretions shut up by malarial poison?—such poison as, if mixed with the animal matter and pent up in a city, would generate regular yellow fever—not contagious, but dependent upon a true malarial cause.

